# Supplementary figures and images for: Imaging Real-Time Tactile Interaction With Two-Person Dual-Coil fMRI
Source: Front Psychiatry. 2020 Apr 28;11:279. doi: 10.3389/fpsyt.2020.00279 (PMC7198901; doi:10.3389/fpsyt.2020.00279)

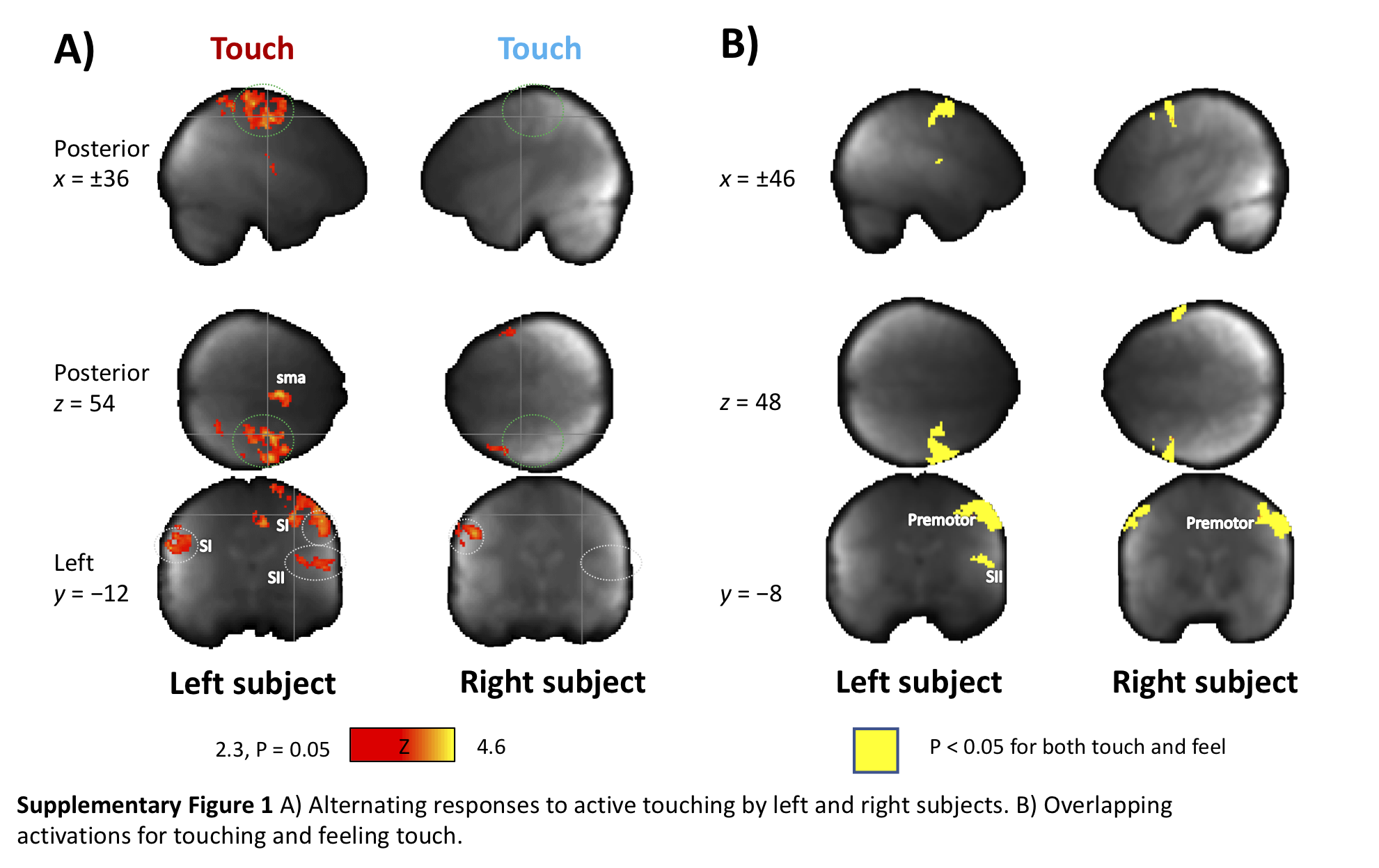

Supplement: Figure S1 — (A) Alternating responses to active touching by left and right subjects. (B) Overlapping activations for touching and feeling touch. [file Image_1.tiff]
